# Proteomic Biomarkers of Preterm Birth Risk in Women with Polycystic Ovary Syndrome (PCOS): A Systematic Review and Biomarker Database Integration

**DOI:** 10.1371/journal.pone.0053801

**Published:** 2013-01-29

**Authors:** Nicolas Galazis, Nikolina Docheva, Kypros H. Nicolaides, William Atiomo

**Affiliations:** 1 Division of Human Development, School of Clinical Sciences, University of Nottingham, Nottingham, United Kingdom; 2 Harris Birthright Research Centre, Kings College Hospital, London, United Kingdom; 3 Department of Fetal Medicine, University College Hospital, London, United Kingdom; John Hunter Hospital, Australia

## Abstract

**Background:**

Preterm Birth (PTB) is a major cause of neonatal mortality and morbidity. Women with Polycystic Ovary Syndrome (PCOS) are at high risk of PTB. There is a need for research studies to investigate the mechanisms linking PCOS and PTB, to facilitate screening, and develop novel preventative strategies.

**Objective:**

To list all the proteomic biomarkers of PTB and integrate this list with the PCOS biomarker database to identify commonly expressed biomarkers of the two conditions.

**Search Strategy:**

A systematic review of PTB biomarkers and update of PCOS biomarker database. All eligible published studies on proteomic biomarkers for PTB and PCOS identified through various databases were evaluated.

**Selection Criteria:**

For the identification of the relevant studies, the following search terms were used: “proteomics”, “proteomic”, “preterm birth”, “preterm labour”, “proteomic biomarker” and “polycystic ovary syndrome”. This search was restricted to humans only

**Data Collection and Analysis:**

A database on proteomic biomarkers for PTB was created while an already existing PCOS biomarker database was updated. The two databases were integrated and biomarkers that were co-expressed in both women with PCOS and PTB were identified and investigated.

**Results:**

A panel of six proteomic biomarkers was similarly differentially expressed in women with PTB and women with PCOS compared to their respective controls (normal age-matched women in the case of PCOS studies and women with term pregnancy in the case of PTB studies). These biomarkers include Pyruvate kinase M1/M2, Vimentin, Fructose bisphosphonate aldolase A, Heat shock protein beta-1, Peroxiredoxin-1 and Transferrin.

**Conclusions:**

These proteomic biomarkers (Pyruvate kinase M1/M2, Vimentin, Fructose bisphosphonate aldolase A, Heat shock protein beta-1, Peroxiredoxin-1 and Transferrin) can be potentially used to better understand the pathophysiological mechanisms linking PCOS and PTB. This would help to identify subgroups of women with PCOS at risk of PTB and hence the potential of developing preventative strategies.

## Introduction

Polycystic ovary syndrome (PCOS) is a complex disorder with reproductive and metabolic consequences including infertility, oligomenorrhoea, hirsutism, acne, hyperandrogenaemia, obesity and an increased risk of hypertension, insulin resistance and Type 2 diabetes in later life [1]–[3]. Women with PCOS are also at increased risk of developing obstetrics complications including pre-eclampsia, gestational diabetes and preterm birth (PTB) [4]–[7]. A recent systematic review showed that pregnant women with PCOS were at least 2 times more likely to give birth prematurely (i.e. before the 37^th^ of gestation) compared to controls (4).

However, the pathophysiological mechanisms underpinning the link between PCOS and PTB are not determined yet.

Various aetiologies have been suggested including the increased incidence of multiple pregnancies and nulliparity [7]. However, when these factors were accounted for and eliminated in recent meta-analyses, pregnant women with PCOS had still increased risk of giving birth prematurely [4]. The pathophysiological mechanisms involved in PTB in women with PCOS are not completely understood but it might be possible that the associated raised estrone levels, hyperinsulinaemia and the subsequent diabetic and hypertensive predispositions may act as co-factors [4], [6].

PTB, defined as birth before the 37^th^ week of gestation, is responsible for 75% of all neonatal deaths and over half the neurological handicap in children [8]–[10]. Despite the advances in antenatal care and the availability of routine screening tests, the rate of PTB has not decreased in the past 30 years [11], mainly because of failure to identify the high-risk groups.

Proteomics is an emerging discipline which involves a large-scale study of the structure and function of proteins allowing the researcher to define protein expression changes in a single experiment [12]. An initial search of the literature through MEDLINE, EMBASE and Cochrane databases using the terms: “proteomics”, “proteomic”, “preterm labour”, “preterm birth”, and “PCOS” or “polycystic ovary syndrome”; no studies were identified where proteomic biomarkers for PTB had been specifically investigated in women with PCOS. However, there were studies where proteomic techniques had been used in the study of PTB and studies where proteomic approaches had been applied to women with PCOS. The aim of this study was therefore to systematically review the research undertaken in PTB using proteomic methodologies to create a database of potential biomarkers of PTB. By integrating this database with an already published database of PCOS biomarkers [13], we aimed to identify any biomarkers that were similarly expressed in both women with PCOS and PTB. Any biomarker common to both conditions would be investigated further.

## Methods

Patient contact was not involved in this study hence Institutional Review Board approval was not necessary.

### Studies Eligible for Review

MEDLINE, EMBASE and Cochrane (registered clinical trials) databases were searched using the terms “proteomics”, “proteomic” and “preterm birth” or “preterm labour”. Animal studies, those which applied proteomics to different PTB groups (eg with intra-amniotic inflammation, without inflammation etc) without comparing them to a normal-term group (the control) or which presented their results as peaks and not as named proteins were excluded.

### Data Abstraction

**Figure 1:**
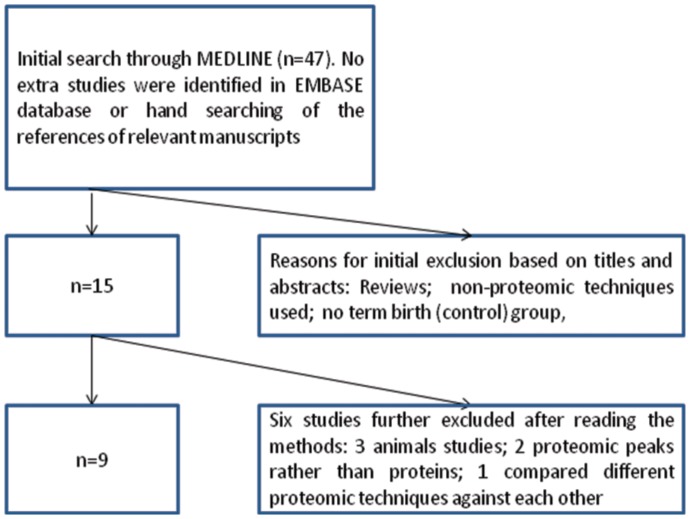
A flow chart summarizing the selection process of the primary studies where proteomic methodologies were used for the identification of biomarkers in PTB.

The original PDFs of studies obtained from the search were located through direct online links to the files from the search results. A manual search of references from all the studies was also conducted to identify any other potentially relevant studies. The search ended in March 2012. The search findings were independently conducted by 2 of the authors (NG and ND). This process is also presented in Figure 1.

### The Main Characteristics of the Studies

The selected studies were screened and specific study characteristics were recorded. These included: number of participants (N), type of proteomic technique used, type of sample collected in each study (eg amniotic fluid) and the selection criteria used. Finally, a list of proteins differentially expressed in women with PTB versus controls (term birth) was created (Table 1). Proteins identified in 2 or more of the primary studies are further listed on Table 2. To minimize selection bias, screening of the studies was independently performed by 2 of the co-authors after agreeing on the selection criteria (NG and ND).

**Table 1:** The main characteristics of each study with the proteins affected in patients in PTL compared to normal individual.

Study	Main Objective	Population		Selection Criteria		Biomarkers [Change (↑/↓)]			Proteomic Technique(s)	Site of Sample	Assessment of Quantitative Data
		N	Mean Age ± (SD) & Age Range	Inclusion	Exclusion	PTB with IAI vs Term birth with IAI	PTB without IAI vs Term birth	PTB with IAI vs PTB without IAI			
Buhimschi et al., 2005 (26)	To identify the proteomic profile of IAI.AF samples from women presenting with PTL	38 21 with preterm delivery;17 delivery at term	Preterm delivery: 27.0 (6.8)Delivery at term: 27.3 (7.5)	Definition of PTL: At least 3 uterine contractions in 10 minutes or 2–3 cm cervical dilatation <37 weeks or PPROMPTB group: WBC >100 cells/mm3 and positive AFCTerm Birth: >37 weeks, WBC <100 cells/mm^3^, -ve AFC	Not Stated	↑- neutrophil defensin-1, neutrophil defensin-2, calgranulin A, calgranulin C	N/A	N/A	SELDI TOF followed by WB	AF	Data were tested for normality using the Kolmogorov–Smirnov test and compared with one-way ANOVA followed by Dunnet’s tests (parametric) or Kruskal–Wallis ANOVA on ranks followed by Dunn’s tests (non-parametric).Receiver–operating characteristic (ROC) curve analysis, intra- and inter-rater kappa calculations were performed using MedCalc (Broekstraat, Belgium) and SPSS (Jandel Scientific, Chicago, Illinois) statistical softwares.
Gravett et al., 2004 (27)	Identify peptide biomarkers for occult/subclinical IAI in women presenting with PTLAF sample from women presenting with PTL between 22 and 34 weeks with intact fetal membranes	33 women Preterm delivery (≤35 weeks):N -11 with IAI, N -11 without IAIDelivery at term (>35 weeks):N-11	Preterm delivery: with IAI 24.5 (5.4), without IAI 26.6 (9.0) Delivery at term: 25.6 (6.0)	PTL: regular uterine contractions at 10 min or less.+either cervical change or cervical dilatation >1 cm or effacement >50% Subclinical IAI: positive AF microbial cultures and/or AF IL-6 concentration >2 ng/ml; chorioamn ionitis	Cervical dilatation >4 cm or ruptured membranes at admission.	All proteins ↑- Both AF and maternal serum: calgranulin B, IGFBP-1 Proteolytic Fragment In AF only: Azurocidin, Macrophage capping protein, Neutrophil gelatinase-associated lipocalin, Myeloperoxidase precursor, L-plastin (lymphocyte cytosolic protein 1), Antibacterial protein Fall-39precursor, Gp-340 variant protein, Novel protein similar to mouse von Ebner salivary gland protein, isoform 2, Leukocyte elastase inhibitor, Calgranulin A	N/A	N/A	SELDI TOF followed by WB LC-MS/MS analysis	AF Maternal serum	Comparison between the 3 groups of women was made using 1-way analysis of variance for continuous data and by the Pearson χ^2^ or 2-tailed Fisher exact test for categorical data. All analyses were performed using SAS, version 8 (SAS Institute Inc, Cary, NC).Using the Pearson χ^2^ statistics and 33 patients allocated equally into the 3 patient groups
Ruetschi et al., 2005 (28)	To identify the proteomic profile of IAI.AF samples from women presenting with PTL	14 PTL with IAI that delivered <34 weeks: N = 7PTL without IA that delivered >34 weeks: N = 7	PTI. with IAI:29(24–31) PTI without IAI: 25(19–31),	Females with singleton pregnancies with PTL <34 weeks. IAI: IL-6 level ≥1.5 ng/ml in PTL PTL = defined as regular uterine contractions (at least 2 uterine contractions/10 min during g 30 min) incombination with cervical changes	Not Stated	N/A	N/A	↑ HNP-1, HNP-2,HNP-3, calgranuli n A, calgranuli n B	SELDI TOF followed by WBLC-MS/MS analysis	AF	The Mann-Whitney test (non-parametric) was used on normalized peak intensities to calculate single marker statistics for the comparison of IAI versus non-IAI. Quantitative variables in Table 1 were analyzed with Mann-Whitney test (non-parametric). The MR score was calculated using Signal-to-Noise (S/N) values for 4 specific peaks. The 4 cutoff values for the Boolean indicators were established using the mean S/N values+2 SD for all non-IAI samples.
Cobo et al., 2009 (29)	Prospective Cohort StudyTo assess proteomic biomarkers and IL-6 alone or in combination to predict IAI, preterm labour, and neonatal morbidity in PTL with intact membranesWomen presenting with PTL between 22 and 36 weeks	86	Negative proteomic biomarkers N = 70: 28.5(6.4)Positive proteomic biomarkers N = 16: 32.5(7.4)	Pregnant females with clinical symptoms of PTL and intact membranes (gestation age 22–36 weeks)	Multiple pregnancies, clinical signs of chorioamnionitis at admission	N/A	N/A	↑in AF-neutrophil defensin-1, neutrophil defensin-2, calgranulin A, calgranulin C	SDS-PAGE methodology followed by WB	AFMaternal Serum	SPSS 14.0 statistical software (SPSS, Inc, Chicago, IL.) was used for the statistical analyses. Receiver-operator curve (ROC) analysis was used to display the relationship between sensitivity and false-positive (FP) rate (1-specificity) and to choose the best cutoff value for IL-6 to diagnose IAI. Foridentification of significant differences among test performances 2×2 contingency tables, χ^2^ test or Fisher exact test analysis of independence were used. Univariate and logistic regression were performed to investigate the relationship between proteomic biomarkers and IL-6 and the occurrence of IAI, preterm delivery <37 wks and neonatal composite morbidity. (P>0.05 significant)
Romero et al., 2010 (30)	Cross-Sectional studyTo identify the proteomic profile of IAI. AF samples from women presenting with PTL	75 PTL without IAI, term delivery, N = 26 PTL without IAI, preterm delivery, N = 25PTL with IAI, preterm delivery N = 24:	PTL without IAI, term delivery: 21(16–38)PTL without IAI, preterm delivery: 28(16–45)PTL with IAI, preterm delivery: 23(17–41)	An episode of spontaneous PTL, intact membranesPTL = regular uterine contractions occurring at a frequencyof at least two every 10 min associated with cervical change before 37 weeks	*Not Stated*	↑-S100A8 protein S100-A8, S100A12 protein S100-A12, ELA2 leukocyte elastase precursor,RETN resistin precursor, TMSL3 thymosin-like 3, MPO isoform H17 of MyeloperoxidasePrecursor, DEFA1; LOC653600; LOC728358 neutrophil defensin 1 precursor,UBE2V2 ubiquitin-conjugating enzyme E2variant 2, S100A9 protein S100-A9, CTSG cathepsin G precursor, HIST1H2BL histone H2B type 1-L,CAMP antibacterial protein FALL-39 precursor, MMP9 matrix metalloproteinase-9 Precursor, MYH9 Myosin-9,HIST2H3C; HIST2H3A histone H3.2, PBEF1 isoform 1 of nicotinamide phosphoribosyltransferase, PPIA;LOC653214;LOC654188 peptidylprolylcis-trans isomerase A, ENO1 Isoform a-enolase of a-enolase, FK506 binding protein 12, HIST1H1D histone H1.3,ACTB actin, cytoplasmic 1, PKM2 isoformM1 of Pyruvate kinase isozymes M1/M2, LCP1 plastin-2, HIST1H4E; HIST1H4F; HIST1H4A;	↑- RETN, TMSL3, SLPI, LTF ↓- LTBP1, OGN	N/A	iTRAQ, SCX chromatography column, HPLC, LC-MS/MS analysis	AF	Mann-Whitney rank sum test was used to compute P values (P<0.05 significant).BiNGO (version 2.0) was used to calculate gene ontology (GO) termenrichment, and CytoScape (version 2.5.1) to visualize the resulting network of GO biological processes.Conversion of protein references to corresponding gene names was done using IPIhumandatabase version 3.35 (redundant gene references were removed to avoid bias). Distributions for statisticalsignificance were tested using the hypergeometric test (equivalent to an exact Fisher test), and the Benjamini and Hochbergcorrection method for false discovery rate (FDR) was applied.
						HIST1H4K; HIST1H4C; HIST1H4L; HIST2H4A; HIST1H4D; HIST2H4B; HIST1H4H; HIST1H4B; HIST1H4I; HIST1H4J; HIST4H4 histone H4,CFL1 cofilin-1, LTF growth-inhibiting protein 12, PGLYRP1 peptidoglycan recognition protein precursor, FCGR3A low affinity immunoglobulin-g-Fc region receptor III-A precursor, ACTN1 a-actinin-1, TPM3 29 kDa protein, TKT transketolase variant (Fragment), YWHAB isoformlong of 14-3-3 protein b/a,LCN2 neutrophil gelatinase-associated lipocalin precursor, MDH1 malate dehydrogenase cytoplasmic, CAPG macrophage-capping protein, ITGAM integrin a-M precursor,VIM vimentin, CHI3L1 chitinase-3-like protein 1 precursor, MIF macrophage migration inhibitory factor, TPI1 triosephosphate isomerase 1 variant, TIMP1 Metalloproteinase inhibitor 1Precursor, SERPINB1 leukocyte elastase inhibitor, GSTP1 glutathione S-transferase P,BASP1 brain acid soluble protein 1, YWHAZ 14-3-3 protein zeta/delta, RAC2 Ras-related C3 botulinum toxin
						substrate 2 precursor, GSTO1 glutathione transferase omega-1, SFTPA1 similar to Pulmonary surfactant associated protein A1 precursor, CALM2; CALM1; CALM3 calmodulin, HSPA1B; HSPA1A heat shock 70 kDa protein 1B,ALDOA fructose-bisphosphate aldolase A, PGD 6-phosphogluconate dehydrogenase decarboxylating,ARHGDIA Rho GDP-dissociation inhibitor 1, MRLC2 myosin regulatory light chain, TXN thioredoxin,PDIA3 protein disulfide-isomerase, CAT catalase, MSN moesin, PGK1 phosphoglycerate kinase 1, PFN1 profilin-1, CTSL1 cathepsin L precursor, HSPA5 proteinhCG_2015269 similar to phosphoglycerate mutase 1 (phosphoglycerate mutase isozyme B) (PGAM-B) (BPGdependent PGAM1) isoform 1, FKBP1A FKBP1A protein, ACTN4a-actinin-4, NME1 nucleoside diphosphate kinase A, YWHAG 14-3-3 protein-g, PIGR polymeric-immunoglobulin receptor precursor, FLNA filamin A a, VCL isoform 1 of Vinculin,					
						MMP8 neutrophil collagenase precursor, YWHAQ 14-3-3 protein theta, KRT19 keratin, type I cytoskeletal 19, LYZ lysozyme C precursor, HSPB1 heat-shock protein b-1, PRDX1 peroxiredoxin-1, PGLS 6-phosphogluconolactonase,↓- HPX hemopexin precursor, COL1A1 collagen a-1(I) chain precursor, OGN mimecan precursor, CPM carboxypeptidase M precursor, PRG2 bone-marrow proteoglycan precursor, ABP1 isoform 2 of Amiloride-sensitive amine oxidase [copper-containing] precursor					
Bujold et al., 2008 (31)	Cross-sectional study To identify the proteomic profile of IAI.AF samples from women presenting with PTL	258 PTL without IAI delivery at term N = 86PTL without IAI delivery preterm N = 86PTL with IAI delivery preterm N = 86	PTL without IAI delivery at term: 23.7±6.6PTL without IAI delivery preterm: 22.9±5.3PTL with IA delivery preterm: 24.6±6.2	Women with PTL and intact membranes (gestation between 20 and 34 weeks)PTL = regular uterine contractions occurring at a frequencyof at least two every 10 min and cervical changebefore 37 weeks	AF RBC count >100 cell	PTB with IAI :↑- Retinol-binding protein, Fibrinopeptide B, Transferrin, MHC class I chain-relatedprotein A (fragment), Transcription elongation factor A protein 2, SRY-box 5, HP8, DSCR2	Delivery at term : ↑- IGFBP-1 precursor (placental protein 12),TPMsk1, von Ebner’s gland protein precursor (tear lipocalin), IL-7 precursor,AMBP, Ribosomal protein S6 kinase alpha-3, APO A1PTB without IAI*: ↑- Retinol-binding protein	N/A	2D-CF and analysis, followed by RP-HPLC SDS-PAGE,MALDI-TOFESI-IT MSLC-MS/MS analysisSELDI-TOF MS Protein Chip immunoassaysELISA for IGFBP-1	AF	Not described
Pereira et al., 2010 (32)	Prospective Cohort To identify the proteins differentially expressed in SPTB compared to term birth.	All 110 PTL but no IAI Pooled sample: N = 5 PTL, N = 5 SPTB	Not Stated	PTL and intact membranes between 20 and 33 weeks and 6 days of gestation.	Not Stated	N/A	↑ in PTB without IAI : Alpha-2-macroglobulin, Plasminogen Complement factor B, Complement	N/A	MALDI-TOF MS 2D LC MS/MS	Serum	Not described
	Serum samples from women presenting with PTL without IAI			PTL = presence of regular uterine contractions that were accompaniedby cervical dilation or effacement at 20 weeks gestation to 33 weeks and 6 days of gestation			component 6, Complement component 8, Complement component 5, Complement component 1,Heparin cofactor 2, Coagulation factor XII, Histidine-rich glycoprotein, Alpha-2-HS-glycoprotein, Angiotensinogen,Sex hormone-binding globulin, ADAM 12, Lipopolysaccharide-binding protein,Alpha-enolase, Pregnancy-specific beta-1- glycoprotein 1, Apolipoprotein B-100, Chorionic somatomammotropinHormone, Pregnancy associated plasma protein A, Gelsolin,Afamin,Hyaluronan-binding protein 2, Beta actin, N-acetylmuramoyl-L-alanineAmidase, Plasma retinol-binding protein, Filamin-A, Tenascin C, Cell adhesion molecule L1-likeProtein, Phosphatidylinositol-glycan specific phospholipase D, Phosphoglycerate mutase 1				
Stella at al., 2009 (33)	To identify the proteins differentially expressed in SPTB compared to term birth.Serum samples from women presenting with PTL without IAI	10 PTL without IAI that delivered at term: N = 5PTL without IAI that delivered at term: N = 5	15–40 years old	Females between 15–40 years old;All pregnant females between 24–41 weeks’ gestation.	Patient refusal; mild/severe preeclampsia, pre-gestational andgestational diabetes and +ve HIV status.	N/A	↑- Fibulin-1, Alpha 1 antitrypsin precursor, sex hormone binding globulin↓- Interalpha-related trypsin inhibitor heavy chain- related protein (IHRP),Complement component 9, Plasma kallikrein B1 precursor	N/A	MALDI-TOF SELDI-TOF 2DE	Serum	Mann-Whitney rank-sum analysis was performed on peaks intensity differences (P<0.05 significant).Measuring specificity and sensitivity for a protein peak in the identification ofPTL by receiver operating characteristic (ROC) curve and calculation of the area under the curve (AUC).Analysis of variance (ANOVA) and post-hoc Tukey’s analysis were used for 2DE. A multivariateANOVA (MANOVA) was carried out on the most significant spots between the groups. The difference in gestational age of serum collection between PLTD and PLPTD was examined with Student *t* test.
Buhimschi et al., 2007 (34)	To identify the proteomic profile of IAI.AF samples from women presenting with PTLComparison of four proteomic biomarkers (MR score) to previously established and proposed markers of IAI,	169 N = 70 PPROM; N = 99Intact membranes	All- 28(16–46) PPROM- 29(16–46) Intact membranes- 25(17–40)	Singleton pregnancy, symptoms of PTL,advanced cervical dilatation ≥3cm, and/or PPROMPTL = regular uterine contractions associated with advanced cervical dilatation or effacement less than 37-week gestation	Not Stated	N/A	N/A	↑- neutrophil defensin-1,neutrophil defensin-2, calgranulin A, calgranulin C	SELDI-TOF	AF	Sigma Stat, version 2.03 (SPSS andMedCalc (MedCalc Software) were used for the statistical analyses. Testaccuracy, positive predictive values (PPV), negative predictive values (NPV), sensitivity and specificity were measured using receiver operator characteristics (ROC) curves.
											Continuous data were compared with the Student *t* test and one-way analysis of variance (ANOVA) followed by Student-Newman-Keuls test (parametric) or Kruskal-Wallis on ranks followed by the Dunn tests (non-parametric). Significant differences among test performances were identified using 2×2 contingency tables and χ^2^ analysis of independence.P values and odds ratios (ORs) were adjusted for potential influences of gestational age and other parameters using a multiple stepwise linear and logistic regression analyses.(P<0.05 significant)

**Index:**

**(S)PTB = **(Spontaneous) Preterm birth.

**1D-GE**  = 1D gel electrophoresis.

**2D-CF**  = 2D chromatographic fractionation.

**2D-LC**  = 2D liquid chromatography.

**2D-DIGE** = Fluorescence two-dimensional differential gel electrophoresis.

**2D-GE/2DE**  = 2D (gel) electrophoresis.

**AF** = Amniotic fluid.

**AFC** = Amniotic fluid culture.

**AMBP** = Alpha-1-microglobulin/bikunin precursor.

**APO**  = Apolipoprotein.

**CF** = Cervical fluid.

**cLC** = Capillary liquid chromatography.

**CVF** = Cervical-vaginal fluid.

**DSCR2** = Down syndrome critical region protein 2.

**ELISAs** = Enzyme-linked immunosorbent assays.

**EOI-TOFMS** = Electrospray-ionization, time-of-flight mass spectrometry.

**ESI-IT MS** = Electrospray ionization-ion trap mass spectrometry.

**FPLC** = Fast protein liquid chromatography.

**HNP** = Human neutrophil protein.

**HP8** =  Human peptide 8.

**HPLC** = High performance liquid chromatography.

**IAI** = Intra-amniotic infection/inflammation.

**IGFBP-1** =  Insulin-like growth factor binding protein-1.

**IL** = Interleukin.

**ITIH4** =  Inter-alpha-trypsin inhibitor heavy chain 4.

**iTRAQ** = Isobaric tag for relative and absolute quantitation.

**LC-MS/MS** = Liquid chromatography – tandem mass spectrometry.

**LTBP1** =  Latent-transforming growth factor β-binding protein isoform 1L.

**LTF** = Growth-inhibiting protein 12/Lactoferrin.

**MALDI-TOF** = Matrix-assisted laser desorption time-of-flight.

**MHC** = Major histocompatibility complex.

**MRM** = Multiple reaction monitoring.

**MS** = Mass spectrometry.

**N/A** =  Not Applicable.

**N** = Number of participants.

**OGN** = Mimecan precursor.

**PANTHER** = Protein analysis through evolutionary relationships.

**PPROM** = preterm premature/(pre-labour) rupture of membranes.

**PT** = Placental tissue.

**PTB** = Preterm birth.

**PTL** = Preterm labour.

**RBC** = Red blood cell.

**RETN** = Resistin.

**RP-HPLC** = Reversed-phase high performance liquid chromatography.

**SCX chromatography column** = Strong cation exchange column.

**SD** = Standard deviation.

**SDS-PAGE** = Sodium dodecyl sulfate-polyacrylamide gel electrophoresis.

**SELDI TOF** = Surface-enhanced laser desorption ionization time-of-flight.

**SILAP** = Stable isotope labeled proteome.

**SLPI** = Antileukoproteinase.

**SRY** = Sex determining region Y.

**TMSL3** =  Thymosin-like 3.

**TPMsk1** =  Tropomyosin sk1 fragment.

**WB** = Western nlotting.

**WBC** = White blood cells.

**Table 2:** The proteins affected most frequently in the studies of women with PTB against women without PTB.

Proteins	Frequency	Study	Main Characteristics of each study
Neutrophil defensin-1 (precursor) (HNP-1)	5/9	Buhimschi et al., 2005 (26)	↑(PTB+IAI), AF, SELDI-TOF followed by WB
		Romero et al., 2010 (30)	↑(PTB+IAI co), AF, iTRAQ, SCX chromatography column, HPLC, LC-MS/MS analysis
		Ruetschi et al., 2005 (28)	↑(PTB+IAI), AF, SELDI-TOF followed by WB, LC-MS/MS analysis
		Cobo et al., 2009 (29)	↑(PTB+IAI), AF, SDS-PAGE methodology followed by WB
		Buhimschi et al., 2007 (34)	↑(PTB+IAI), AF, SELDI-TOF followed by WB
Neutrophil defensin-2 (precursor) (HNP-2)	4/9	Buhimschi et al., 2005 (26)	↑(PTB+IAI), AF, SELDI-TOF followed by WB
		Ruetschi et al., 2005 (28)	↑(PTB+IAI), AF, SELDI-TOF followed by WB, LC-MS/MS analysis
		Cobo et al., 2009 (29)	↑(PTB+IAI), AF, SDS-PAGE methodology followed by WB
		Buhimschi et al., 2007 (34)	↑(PTB+IAI), AF, SELDI-TOF followed by WB
Calgranulin A (S100-A8)	6/9	Buhimschi et al., 2005 (26)	↑(PTB+IAI), AF, SELDI-TOF followed by WB
		Ruetschi et al., 2005 (28)	↑(PTB+IAI), AF, SELDI-TOF followed by WB, LC-MS/MS analysis
		Gravett et al., 2004 (27)	↑(PTB+IAI), AF and maternal serum, SELDI-TOF followed by WB, LC-MS/MS analysis
		Romero et al., 2010 (30)	↑(PTB+IAI), AF, iTRAQ, SCX chromatography column, HPLC, LC-MS/MS analysis
		Cobo et al., 2009 (29)	↑(PTB+IAI), AF, SDS-PAGE methodology followed by WB
		Buhimschi et al., 2007 (34)	↑(PTB+IAI), AF, SELDI-TOF followed by WB
Calgranulin B (S100-A9)	4/9	Gravett et al., 2004 (27)	↑(PTB+IAI), AF and maternal serum, SELDI-TOF followed by WB, LC-MS/MS analysis
		Ruetschi et al., 2005 (28)	↑(PTB+IAI), AF, SELDI-TOF followed by WB, LC-MS/MS analysis
		Romero et al., 2010 (30)	↑(PTB+IAI), AF, iTRAQ, SCX chromatography column, HPLC, LC-MS/MS analysis
		Pereira et al., 2010 (32)	↑ (PTB+IAI) in SPTB, Serum, MALDI-TOF MS, 2D LC MS/MS
Calgranulin C (S100-A12)	4/9	Buhimschi et al., 2005 (26)	↑(PTB+IAI), AF, SELDI-TOF followed by WB
		Cobo et al., 2009 (29)	↑(PTB+IAI), AF, SDS-PAGE methodology followed by WB
		Romero et al., 2010 (30)	↑(PTB+IAI), AF, iTRAQ, SCX chromatography column, HPLC, LC-MS/MS analysis
		Buhimschi et al., 2007 (34)	↑(PTB+IAI), AF, SELDI-TOF followed by WB
IGFBP-1(proteolytic fragment precursor)	3/9	Gravett et al., 2004 (27)	↑(PTB+IAI), AF and maternal serum, SELDI-TOF followed by WB, LC-MS/MS analysis
		Bujold et al., 2008 (31)	↑(PTL-IAI and delivery at term), AF, 2D-CF and analysis, followed by RP-HPLC,SDS-PAGE, MALDI-TOF, ESI-IT MS, LC-MS/MS analysis, Liquid-phase (direct) mass spectrometry analysis, SELDI-TOF MS Protein Chip immunoassays, ELISA for IGFBP-1
		Pereira et al., 2010 (32)	↑ (PTL-IAI) in SPTB, Serum, MALDI-TOF MS, 2D LC MS/MS
APO A-1	2/9	Bujold et al., 2008 (31)	↑(PTL-IAI and delivery at term), AF, 2D-CF and analysis, followed by RP-HPLC, SDS-PAGE, MALDI-TOF, ESI-IT MS, LC-MS/MS analysis, Liquid-phase (direct) mass spectrometry analysis, SELDI-TOF MS Protein Chip immunoassays, ELISA for IGFBP-1
		Pereira et al., 2010 (32)	↑ (PTL-IAI) in SPTB, Serum, MALDI-TOF MS, 2D LC MS/MS
Retinol-binding protein	2/9	Bujold et al., 2008 (31)	↑(PTL±IAI and preterm delivery), AF, 2D-CF and analysis, followed by RP-HPLC, SDS-PAGE, MALDI-TOF, ESI-IT MS, LC-MS/MS analysis, Liquid-phase (direct) mass spectrometry analysis, SELDI-TOF MS Protein Chip immunoassays, ELISA for IGFBP-1
		Pereira et al., 2010 (32)	↑ (PTL-IAI) in SPTB, Serum, MALDI-TOF MS, 2D LC MS/MS
FLNAFilamin A α	2/9	Romero et al., 2010 (30)	↑(PTB+IAI), AF, iTRAQ, SCX chromatography column, HPLC, LC-MS/MS analysis
		Pereira et al., 2010 (32)	↑ (PTL-IAI) in SPTB, Serum, MALDI-TOF MS, 2D LC MS/MS
Macrophage-capping protein	2/9	Romero et al., 2010 (30)	↑(PTB+IAI), AF, iTRAQ, SCX chromatography column, HPLC, LC-MS/MS
		Gravett et al., 2004 (27)	↑(PTB+IAI), AF and maternal serum, SELDI-TOF followed by WB, LC-MS/MS analysis
Neutrophil gelatinase-associated lipocalin(precursor)	2/9	Romero et al., 2010 (30)	↑(PTB+IAI), AF, iTRAQ, SCX chromatography column, HPLC, LC-MS/MS
		Gravett et al., 2004 (27)	↑(PTB+IAI), AF and maternal serum, SELDI-TOF followed by WB, LC-MS/MS analysis
Myeloperoxidase precursor/MPO isoform H17 of Myeloperoxidase Precursor	2/9	Romero et al., 2010 (30)	↑(PTB+IAI), AF, iTRAQ, SCX chromatography column, HPLC, LC-MS/MS
		Gravett et al., 2004 (27)	↑(PTB+IAI), AF and maternal serum, SELDI-TOF followed by WB, LC-MS/MS analysis
FALL-39(precursor)	2/9	Gravett et al., 2004 (27)	↑(PTB+IAI), AF and maternal serum, SELDI-TOF followed by WB, LC-MS/MS analysis
		Romero et al., 2010 (30)	↑(PTB+IAI), AF, iTRAQ, SCX chromatography column, HPLC, LC-MS/MS
Leukocyte elastase inhibitor (SERPINB1)	2/9	Gravett et al., 2004 (27)	↑(PTB+IAI), AF and maternal serum, SELDI-TOF followed by WB, LC-MS/MS analysis
		Romero et al., 2010 (30)	↑(PTB+IAI), AF, iTRAQ, SCX chromatography column, HPLC, LC-MS/MS
Von Ebner’s gland protein precursor/Novel protein similar to mouse von Ebner salivary gland protein	2/9	Gravett et al., 2004 (27)	↑(PTB+IAI), AF and maternal serum, SELDI-TOF followed by WB, LC-MS/MS analysis
		Bujold et al., 2008 (31)	↑(PTL-IAI and delivery at term), AF, 2D-CF and analysis, followed by RP-HPLC, SDS-PAGE, MALDI-TOF, ESI-IT MS, LC-MS/MS analysis, Liquid-phase (direct) mass spectrometry analysis, SELDI-TOF MS Protein Chip immunoassays, ELISA for IGFBP-1

**Index:**

**(S)PTB** = (Spontaneous) Preterm birth.

**2D-CF**  = 2D chromatographic fractionation.

**AF** = Amniotic fluid.

APO  = Apolipoprotein.

**ELISA** = Enzyme-linked immunosorbent assays.

**ESI-IT MS** = Electrospray ionization-ion trap mass spectrometry.

**HPLC** = High performance liquid chromatography.

**IAI** = Intra-amniotic Infection/Imflammation.

**iTRAQ** = Isobaric tag for relative and absolute quantitation.

**LC MS/MS** = Liquid chromatography – tandem mass spectrometry,

**MALDI-TOF** = Matrix-assisted laser desorption time-of-flight,

**MS** = Mass spectrometry,

**PTB** =  Preterme birth.

**PTL** = Preterm labour,

**RP-HPLC** = Reversed-phase high performance liquid chromatography,

**SCX chromatography column** = Strong cation exchange column,

**SDS-PAGE** = Sodium dodecyl sulfate-polyacrylamide gel electrophoresis,

**SELDI-TOF = **Surface-enhanced laser desorption ionization time-of-flight;

**WB** = Western blotting.

### Methodological Quality Assessment

**Figure 2:**
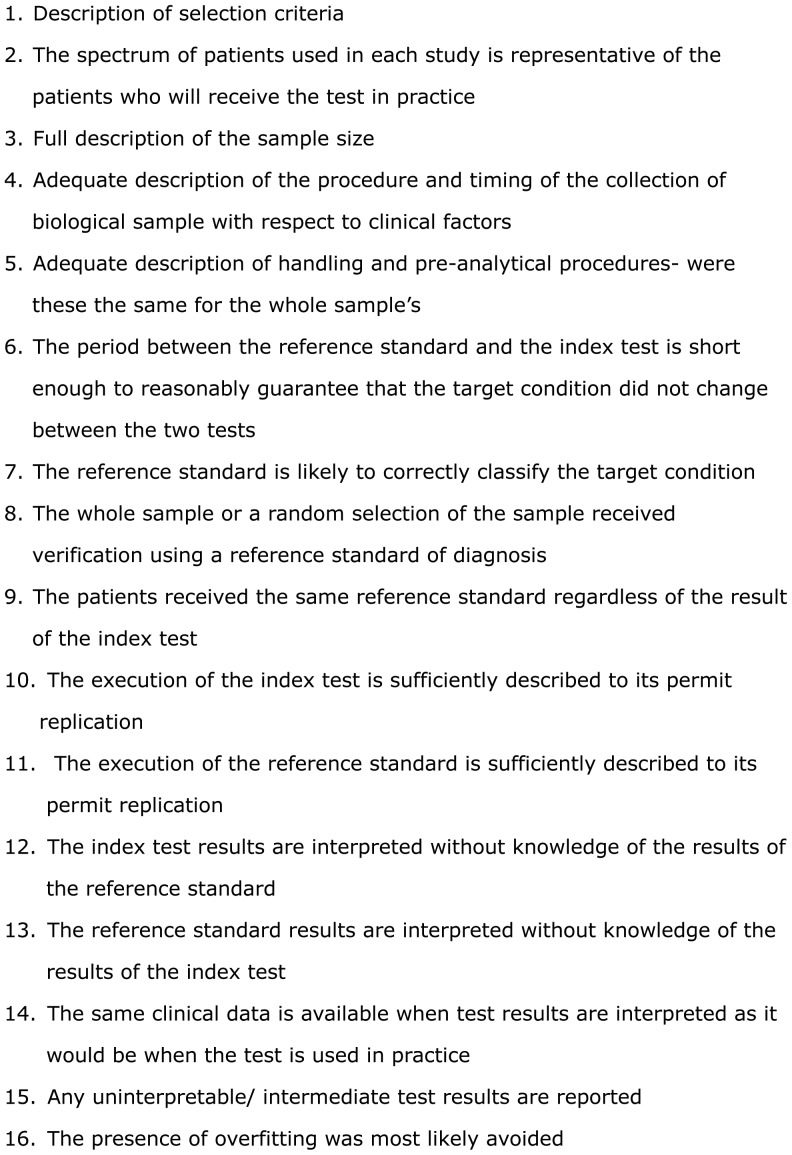
According to QUADOMICS Tool the following methodological criteria were applied to this review.

The methodological quality of primary studies applying proteomics in women with PTB was determined using the QUADOMICS Tool, an adaptation of QUADAS (a quality assessment tool for use in systematic reviews of the diagnostic accuracy studies) which takes into account the particular challenges encountered in “-omics” based techniques (Figure 2) [14]. The methodologies of the studies which achieved 12/16 or more on the QUODOMICS Tool were classified as high quality (HQ) whereas those which scored 11/16 or less were classified as low quality (LQ). This quality assessment was performed independently by two of the co-authors (NG and ND).

### Updating the PCOS Proteomics Database

The methods used to search for and collect the data on the PCOS proteomic database have been previously published and validated [13]. An updated literature search was performed on MEDLINE, EMBASE and the ISI web of knowledge (v4.2) databases using the following search terms ‘polycystic ovary syndrome’ and “proteomic”, “proteomics”, “proteomic biomarker” without any limits/restrictions. All relevant studies published after the original PCOS database were reviewed. Eleven studies [15]–[25] were identified including four reviews and one study on mice. The review articles and the study on mice [18], [23]–[24] were excluded. A further three studies were abstracts from conference proceedings with no primary proteomic data on PCOS so they were also excluded [19]–[21]. The data from the three remaining studies was accessed through direct online links to the files from the search results [15]–[16], [22].

### Integrating the Proteomic Database of PTB with the PCOS Database

Proteomic biomarkers for PTB identified in two or more of the primary studies are listed on Table 2. These were then compared to the updated database of proteomic biomarkers for PCOS. Any commonly expressed biomarkers where indentified. A note was made of their function and of the tissue from which they originated in women with PCOS. Given the limited number of commonly expressed biomarkers identified, this exercise was expanded to all the proteomic biomarkers identified in PTB against the updated PCOS database. This process was independently performed by two of the authors (NG and ND).

## Results

### Proteomic Studies for PTB

Figure 1 demonstrates the selection process of the primary studies where proteomic methodologies were used for the identification of biomarkers of PTB. The initial search conducted through MEDLINE yielded 47 articles which included 7 reviews. After screening the titles and abstracts, 15 primary studies were isolated. Studies were excluded if they were review articles, proteomic techniques were not used or if they did not compare PTB with a term birth (control) group. Three studies involving animals only, 2 presenting proteomic peaks rather than proteins and 1 comparing different proteomic approaches were further excluded leaving 9 primary studies [26]–[34] eligible for this review. Further searches of the Cochrane (registered clinical trials) and EMBASE databases and hand searching of the references of relevant manuscripts did not yield additional articles.

### General Characteristics of the Proteomic Studies Investigating Biomarkers of PTB

A total of 9 studies were identified from the literature (Table 1). The overall number of participants was 820. Sample sites differed between studies; 5 studies used amniotic fluid (AF) only [26], , 2 studies used AF and maternal serum [27], [29] and 2 studies used maternal serum only [32]–[33]. In general, the selection criteria were adequately described. However, 4 studies failed to explicitly state their exclusion criteria [26], [28], [30], [32]. The study population was fully described in 8 studies with only one study not describing the mean age and age range of the patients [32]. Various proteomic techniques were used in the 9 studies with SELDI-TOF (Surface-enhanced laser desorption ionization time-of-flight), MALDI-TOF (Matrix-assisted laser desorption time-of-flight) and LC-MS/MS (Liquid Chromatography – Tandem Mass Spectrometry) being the most common (Table 1).

### Assessing the Quality of the Relevant Studies

Six out of the 9 studies were HQ fulfilling 12 or more of the 16 QUADOMICS criteria [27]–[28], [30]–[31], [33]–[34]. The remaining 3 studies were LQ achieving less than 12 out of the 16 quality criteria [26], [29], [32].

### Determining the Proteins Most Frequently Affected in the PTB Studies

A total of 201 different proteomic biomarkers were identified in the 9 studies, 15 of which were identified in 2 studies or more (Table 2). These included: Neutrophil defensin-1 (precursor) (HNP-1), Neutrophil defensin-2 (precursor) (HNP-2), Calgranulin A (S100-A8), Calgranulin B (S100-A9), Calgranulin C (S100-A12), IGFBP-1 (proteolytic fragment precursor), APO A-1, Retinol-binding protein, FLNA (Filamin A α), Macrophage-capping protein, Neutrophil gelatinase-associated lipocalin (precursor), Myeloperoxidase precursor/MPO isoform H17 of Myeloperoxidase Precursor, FALL-39 (precursor), Leukocyte elastase inhibitor (SERPINB1), and Von Ebner’s gland protein precursor/Novel protein similar to mouse von Ebner salivary gland protein.

### Cross Referencing Proteomic Biomarkers Identified in Primary Studies of PTB in Database of Proteomic Biomarkers for PCOS

Thirty-two additional proteomic biomarkers for PCOS were identified in the process of updating the PCOS proteomic database (available on request) and these were merged with the old database. Some biomarkers were variants of the same protein which was presumed to be due to varied post-translational modifications or splicing variants. A free text search of the PCOS proteomic biomarker database was carried out initially using the 15 PTB biomarkers identified in two or more studies in our systematic review.

This search was then expanded to include the remaining 186 PTB biomarkers identified in the 9 PTB studies. Six biomarkers were similarly over-expressed in women with PTB and with PCOS compared to controls. These biomarkers include Pyruvate kinase M1/M2 (PKM1/M2), Vimentin, Fructose bisphosphonate aldolase A, Heat shock protein beta-1, Peroxiredoxin-1 and Transferrin.

## Discussion

For this review, a biomarker was defined as a characteristic that can be objectively measured and evaluated as an indicator of pathological processes [35]. This study has, for the first time, identified a panel of 6 proteomic biomarkers which were similarly over-expressed in women with PTB and in women with PCOS. These biomarkers include PKM1/M2, Vimentin, Fructose bisphosphonate aldolase A, Heat shock protein beta-1, Peroxiredoxin-1 and Transferrin.

PKM1/M2 was found to be elevated both in patients with PCOS and with PTB. Pyruvate kinase catalyzes the last step of glycolysis where phosphoenolpyruvate (PEP) is converted to ADP. PKM2 is known to interact with a variety of biological molecules such as A-Raf, FGFR-1 and Jak-2 mutant and is also implicated in cancer metabolism [36]. High Pyruvate Kinase activity has been found both in rat and human placentae, indicating that the placenta is having a high glycolytic potential [37]–[38]. This was indeed the case, since further results on placentae in women with gestational diabetes showed increased Pyruvate Kinase activity [39]–[40]. A large meta-analysis involving pregnant women with PCOS demonstrated an increase in the prevalence of gestational diabetes compared to pregnant women without PCOS [6]. It is also well established that women with PCOS have an increased risk of developing Type 2 diabetes compared to the general population. We therefore believe that the increased levels of PKM1/M2 observed in both PCOS and PTB may represent a common defect in glucose metabolism. Fructose Bisphosphonate Aldolase A is a glycolytic enzyme found in all tissues [41]. It acts in the same pathway as PKM1/M2 and thus the increase in both PCOS and PTB can be explained using the above hypothesis.

Vimentin is an intermediate filament (IF) protein which is an important cytoskeletal part of mesenchymal cells. It plays a vital role in anchoring and positioning organelles in the cytosol [42]. Vimentin expression seems to be increased in inflammatory and immunological processes evident in studies involving patients with rheumatoid arthritis and Group A streptococcal infections [43]–[44]. Its increase in both PCOS and PTB is thus justified since both conditions have inflammatory and immunological pathophysiology.

Transferrin is a glycoprotein that transports iron and is known to promote iron transport in the ovarian follicles [45]. Transferrin also plays a crucial role in pregnancy where its expression in the villous syncytiotrophoblasts is significantly increased in women with PTB compared to those with normal pregnancies [46]. Transferrin is a recognized stress/acute phase response molecule. Its increase in both women with PCOS and PTB can be explained on the basis of the inflammatory component of the two conditions.

HSPB1 is also known as HSP27 and HSP28 and its levels are increased by mechanisms such as oxidative stress, heat shock exposure, infection, inflammation and ischemia [47]–[48]. As with Transferrin and Vimentin, the higher expression of HSPB1 observed in both women with PCOS and PTB compared to controls reflect the inflammatory process involved in this conditions.

Peroxiredoxin-1 is involved in antioxidant defense mechanisms, cellular redox reactions, signaling transduction pathways and may have possible chaperone activity [49]. Its over-expression in both PCOS and PTB may represent the differentiating steps of the immune reaction that take place in the two conditions.

We acknowledge that the disparate accuracy and precision of the various quantitative and semi-quantitative techniques could pose a challenge with a combined assessment of the results. This is, however, an issue with all systematic reviews and metanalyses which could be affected by clinical heterogeneity. This was the reason we chose to report differential protein expression as either up- or down-regulated which is consistent with previously published systematic reviews of proteomic biomarkers [50].

A consistently emerging theme from studies using proteomic approaches in PCOS is the potential role of immunoregulation/inflammation and antioxidants in the pathogenesis of the condition. These two pathways have also been implicated in PTB and insulin resistance which are both of concern in women with PCOS [1]–[7]. Using inflammatory factors as biomarkers for disease conditions is challenging as inflammation is associated with a multitude of other pathological conditions. However, this is a limitation that applies to all biomarker studies of complex diseases such as one previously published in this journal and not just inflammatory biomarkers [51]. We do not propose at this stage that the biomarkers identified in our study are used as definitive biomarkers of PTB and PCOS rather that our results inform further mechanistic and validation studies and can be used to better understand the pathophysiological mechanisms linking PCOS and PTB.

Although proteomic and other “-omic” technologies offer a great potential for the generation of new insights into disease aetiology, concerns have been expressed about the relatively slow pace at which research findings have been translated into clinical care [52]–[53]. In addition, proteomic techniques have limited ability in detecting low-abundance proteins, some of which may have diagnostic potential. There has been a call for greater focus on data integration from primary proteomic studies in order to improve translation of research findings and prospective validation [54]. The sample sizes and number of biomarkers identified following these studies runs the risk of false positives and this is a limitation of all biomarker studies [55]. These issues again emphasize the need for collaboration, data synthesis and integration (as done in this review) in order to identify a shortlist of replicated biomarkers which can be validated in subsequent hypothesis-driven research [53]. We therefore see great value in informing the scientific community about these research findings at this stage as in the area of “omic” research, data sharing and collaboration is vital for progress. For example, an independent research group with access to stored tissue samples from women with PCOS who have had PTB may, based on this review, decide to independently validate the biomarkers identified in their cohort which would save time. For improved accuracy, it is essential that the same definition of biomarker and selection criteria are employed by future validation studies.

In summary, by integrating data from proteomic studies in PTB with data from proteomic studies in PCOS, we have for the first time identified a panel of 6 promising biomarkers of PTB in women with PCOS. If validated, these biomarkers could provide a useful framework on which the knowledge base in this area could be developed, and will facilitate future mathematical modeling to enhance screening and prevention of PTB in women with PCOS who have been shown to be at increased risk. A well coordinated multidisciplinary collaboration of basic scientists, clinicians and mathematicians is vital to achieve this goal.
